# Biomechanical and Histological Evaluation of Roughened Surface Titanium Screws Fabricated by Electron Beam Melting

**DOI:** 10.1371/journal.pone.0096179

**Published:** 2014-04-30

**Authors:** Jun Yang, Hong Cai, Jia Lv, Ke Zhang, Huijie Leng, Zhiguo Wang, Zhongjun Liu

**Affiliations:** Department of Orthopedics, Peking University Third Hospital, Haidian District, Beijing, China; INSERM U1059/LBTO, Université Jean Monnet, France

## Abstract

**Background:**

Various fabrication methods are used to improve the stability and osseointegration of screws within the host bone. The aim of this study was to investigate whether roughened surface titanium screws fabricated by electron beam melting can provide better stability and osseointegration as compared with smooth titanium screws in sheep cervical vertebrae.

**Methods:**

Roughened surface titanium screws, fabricated by electron beam melting, and conventional smooth surface titanium screws were implanted into sheep for 6 or 12 weeks (groups A and B, respectively). Bone ingrowth and implant stability were assessed with three-dimensional imaging and reconstruction, as well as histological and biomechanical tests.

**Results:**

No screws in either group showed signs of loosening. Fibrous tissue formation could be seen around the screws at 6 weeks, which was replaced with bone at 12 weeks. Bone volume/total volume, bone surface area/bone volume, and the trabecular number were significantly higher for a define region of interest surrounding the roughened screws than that surrounding the smooth screws at 12 weeks. Indeed, for roughened screws, trabecular number was significantly higher at 12 weeks than at 6 weeks. On mechanical testing, the maximum pullout strength was significantly higher at 12 weeks than at 6 weeks, as expected; however, no significant differences were found between smooth and roughened screws at either time point. The maximum torque to extract the roughened screws was higher than that required for the smooth screws.

**Conclusions:**

Electron beam melting is a simple and effective method for producing a roughened surface on titanium screws. After 12 weeks, roughened titanium screws demonstrated a high degree of osseointegration and increased torsional resistance to extraction over smooth titanium screws.

## Introduction

Titanium alloy screws, such as anterior plate screws and pedicle screws, are routinely used in orthopedics to maintain the stability of an implant. However, screw loosening, such as aseptic loosening, which is caused by a weakened interface between the screw surface and the surrounding bone, results in fixation failure [1]–[3], with potentially severe complications for the patient [4]. It is now widely accepted that the surface topography of titanium alloy screws plays a fundamental role in the attachment, proliferation, and differentiation of osteoblasts at the repair site, all of which is important for the successful osseointegration of the screw with the host bone. Many studies show that titanium alloy screws with a roughened surface offer good cell attachment and differentiation. Anselme et al. observed higher levels of adhesion and differentiation of osteoblasts on roughened surfaces than on smooth and polished surfaces [5], and other studies have also confirmed that higher numbers of osteoblast adhere to roughened surfaces than smooth surfaces [6], [7].

Various methods for fabricating rough-surfaced titanium alloy implants have been considered. Jenny et al. compared osteoclast activity on smooth, acid-etched, or sandblasted acid-etched titanium. They found similar osteoclast characteristics on the rough titanium surfaces but reduced osteoclast activity on smooth surfaces [8]. Yang and co-workers [9] treated porous titanium with H_2_O_2_/TaCl_5_ or H_2_O_2_/TaCl_5_ plus a subsequent incubation in simulated body fluid. They then investigated the effect of surface treatments on the attachment and differentiation of mesenchymal stem cells (MSCs). Their results suggest that a rough surface has a greater potential for promoting MSC differentiation along the osteogenic lineage. Thereafter, further evidence suggests that a rough or microporous structure endows titanium implants with osteoinductive properties [10]–[12].

Studies also illustrate that titanium alloy screw stability can be augmented by changing its shape. Wan and co-workers [13] compared the biomechanical and histological properties of expandable pedicle screws (EPSs) and standard pedicle screws in osteoporotic spines of sheep. They found that EPSs resulted in better biomechanical and histological properties as compared with standard screws. Others [14], [15] have reported similar findings, showing that EPSs offer a 30% increase in bone pullout strength as compared with conventional pedicle screws.

To prevent screw loosening, chemical or physical methods can be used to modify the screw surface or improve local stability. However, the use of chemical reagent to adjust the properties of screws has limited use in clinical applications. In this study, we sought to investigate improvements in the biomechanical and histological properties of surface titanium screws roughened using electron beam melting (EBM) in sheep cervical spine.

## Materials and Methods

### Screws

**Figure 1:**
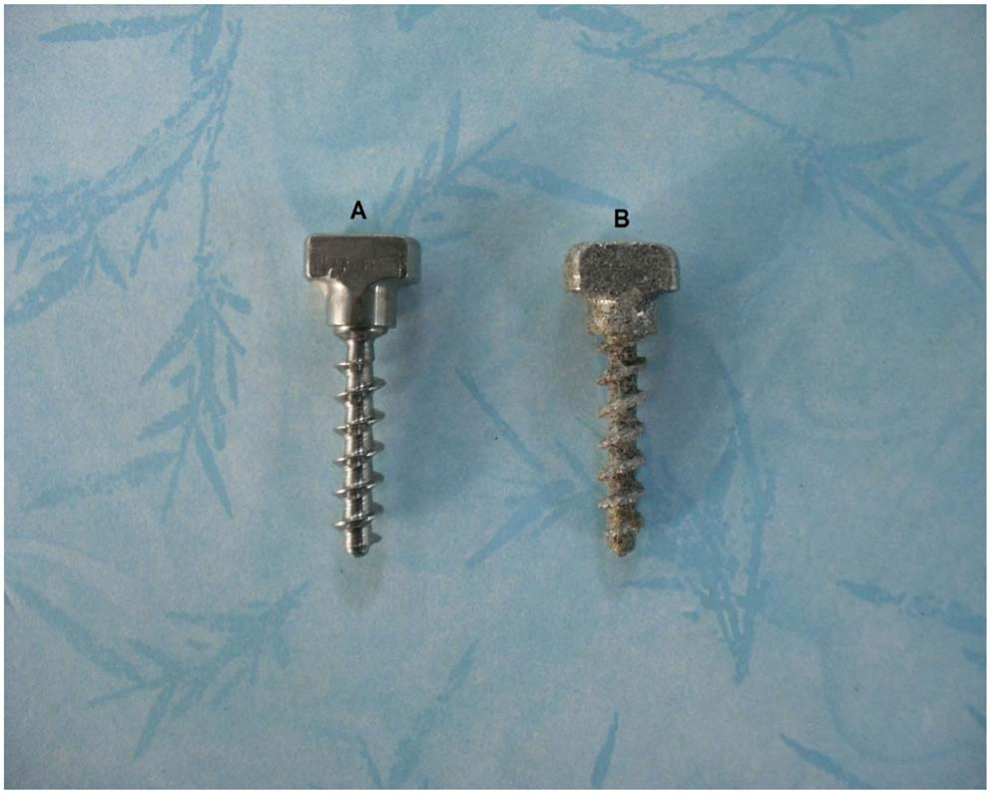
(A) Conventional titanium alloy screw with a smooth surface (SS). (B) Titanium alloy screws with a roughened surface (RS) were fabricated by electron beam melting (EBM).

Two experimental groups were created: an experimental group (n = 16), comprising titanium alloy screws with a roughened surface (RS), and a control group (n = 16), comprising conventional titanium alloy screws with a smooth surface (SS). All screws had an outer diameter of 5 mm and a length of 25 mm (Fig. 1). Screws in the RS group were fabricated by EBM S12 system (Acram AB, Sweden). Briefly, titanium alloy powder (Ti6Al4V, particle size 45–100 um) was melted in a layer-by-layer fashion in the EBM S12 system and the excess powder was removed from the implant. A scanning electron microscope (SEM) was used to study the micro-structural surface characteristics.

### Study Design

This study was approved by the animal ethics committee of the Peking University Health Science Center (Approval Number: LA2013-71).

**Figure 2:**
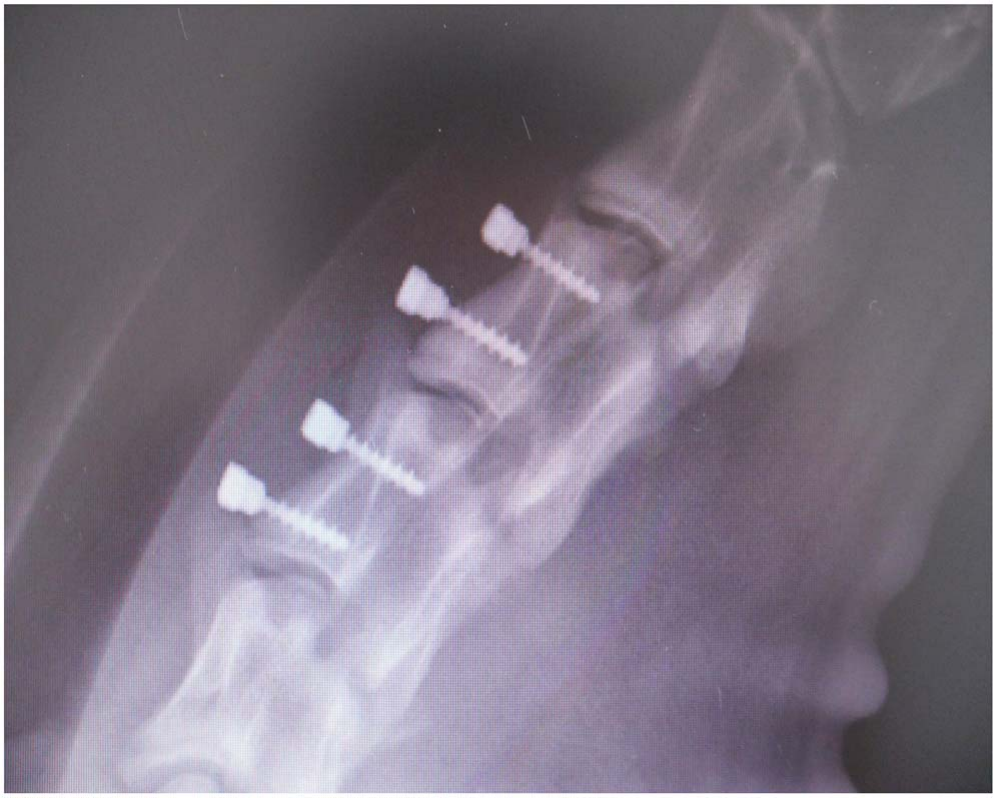
The surgical sites where the screws were placed.

Eight adult male Small Tail Han Sheep (48.1±5.8 kg; 18±5 months of age) were used in this study. The sheep were anesthetized and placed in a supine position. A vertical incision was made at approximately C3 and C4 level to expose the anterior cervical vertebral elements. Using a high-speed burr, two platforms with a diameter of 5 mm were ground on the C3 and C4 vertebral bodies. An electric drill was then used to create a uniform 3-mm pilot hole into which four screws (2 RS and 2 SS) were implanted (one RS and one SS per vertebral body) (Fig. 2). Four screws (2 RS and 2 SS) were harvested for histological examination and 12 screws (6 RS and 6 SS) for biomechanical testing followed by micro-CT at 6 and 12 weeks (*n* = 8 at each time point).

### Histology

Specimens were harvested, fixed in 10% formalin for 2 weeks, dehydrated in graded ethanol (40%, 75%, 95%, and 100%) for 2 days under vacuum, and then embedded in methylmethacrylate. Thick slices (150–300 µm) were cut from the blocks and ground to a thickness of 40–50 µm using transverse saw cuts and a polishing machine (Exact Band Saw; Exact Apparatebau, Norderstedt, Germany). Each specimen were cut 20 to 30 sections for analysis and sections were stained with toluidine blue.

### Three-dimensional Imaging and Reconstruction

Specimens were dissected into cylinders of 20 mm in diameter, using the screw as the center point, for scanning with an X-ray micro-tomography system scanner (Inveon, Siemens Medical Solutions Inc. Malvern, PA, USA). Briefly, cut specimens were placed vertically onto the sample holder with the long axis of the implant perpendicular to the scanning beam. The samples were then scanned at a resolution of 26.1 µm and serial images of the complete construct were obtained. An equal-sized region of interest (ROI) surrounding the RS and SS screws was constructed and all images were analyzed under the same thresholding conditions.

### Mechanical Testing

Specimens were kept frozen at −20°C in two plastic bags until biomechanical testing. The specimens were thawed overnight at 6°C according to the method described by Panjabi et al. [16], and were then embedded in polymethylmethacrylate. Mechanical testing was performed using a servohydraulic materials testing machine (MTS 858 Bionix machine, MTS System Inc., Minneapolis, MN, USA).

For pullout tests, extraction tension was applied a rate of 0.2 mm/s over a total distance of 10 mm. From the pullout tests, the stiffness (N/mm) and axial force (N) of each screw were calculated. The peak pullout load was defined as the highest load that the bone–screw interface could resist before failure.

For the torsion tests, screws were rotated 30° counter-clockwise at a speed of 0.5°/s. From the torsion tests, the maximum torque (Nmm) and the angle-related stiffness (Nmm/°) were calculated.

## Statistical Analysis

Data analysis was performed using SPSS Version 18.0 (Chicago, IL, USA). Results are depicted as means ± standard deviations. For the three-dimensional image analysis, the parameters were compared using the Mann-Whitney U test. Student’s two-tailed t-test was applied to compare of pullout and torque data. Differences were deemed to be significantly different when *P*<0.05.

## Results

### Screws

**Figure 3:**
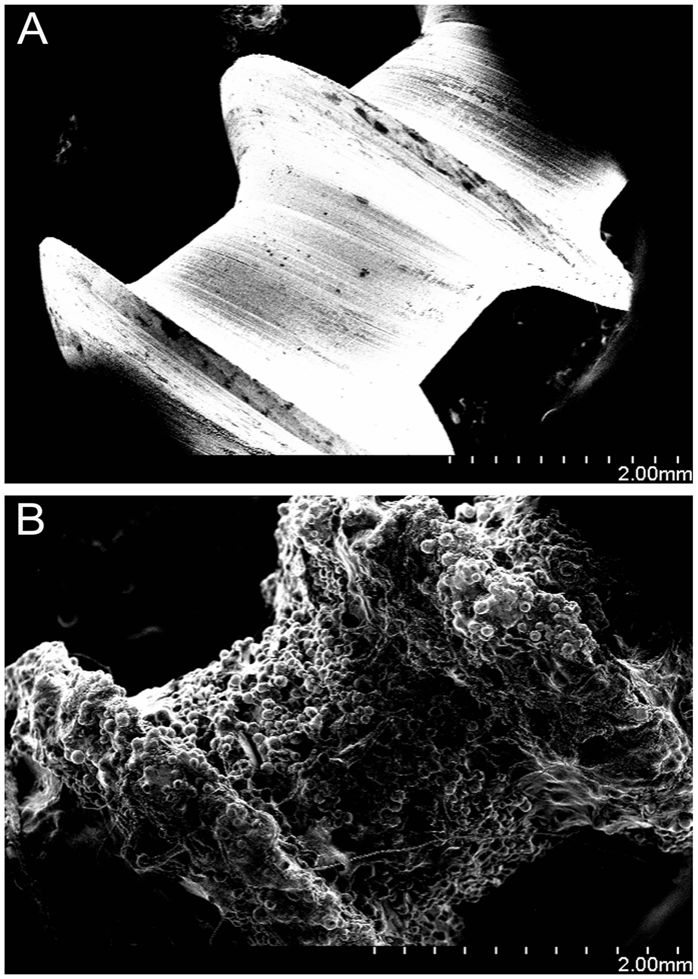
Scanning electron microscopy images of (A) smooth surface (SS) screws and (B) roughened surface (RS) screws. The melted Ti6Al4V powder can be observed clearly in Fig. 3B.

SEM images of the screw surface microstructure for RS and SS screws are depicted in Figure 3. The roughness of the RS screw surface can be seen as well as the melted Ti6Al4V powder (Fig. 3B).

### Histology

**Figure 4:**
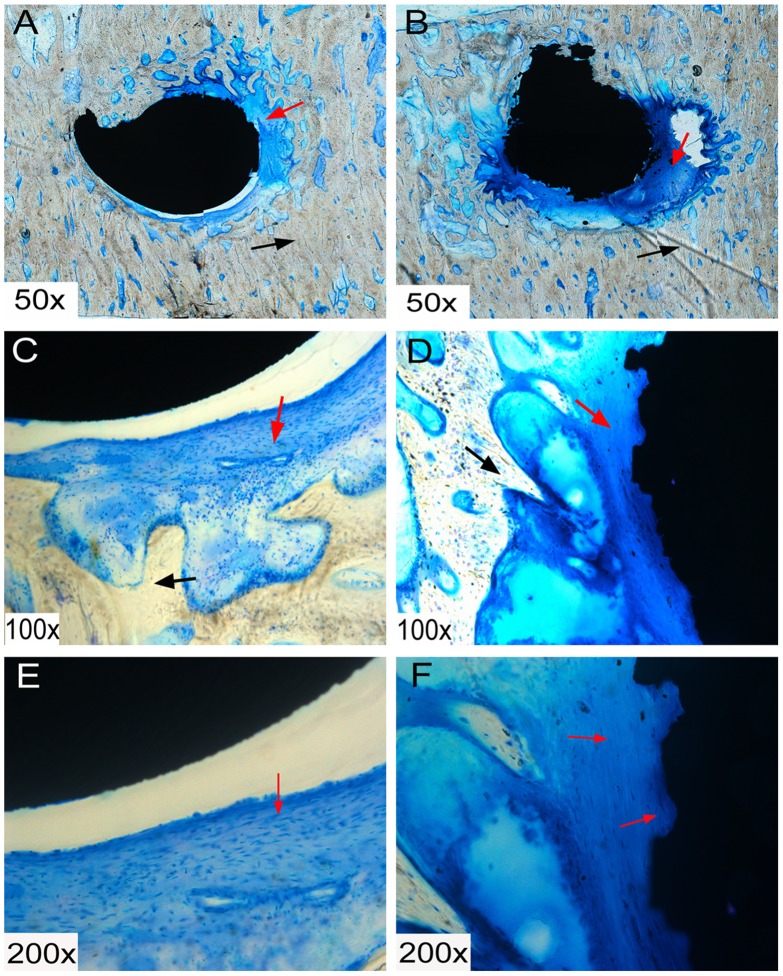
Histological sections of smooth surface (SS) (A, C and E) and roughened surface (RS) (B, D and F) screws at 6 weeks. Fibrous tissue formation can be seen around the screws, as indicated by the red arrow. Black arrows indicate the bone tissue.

**Figure 5:**
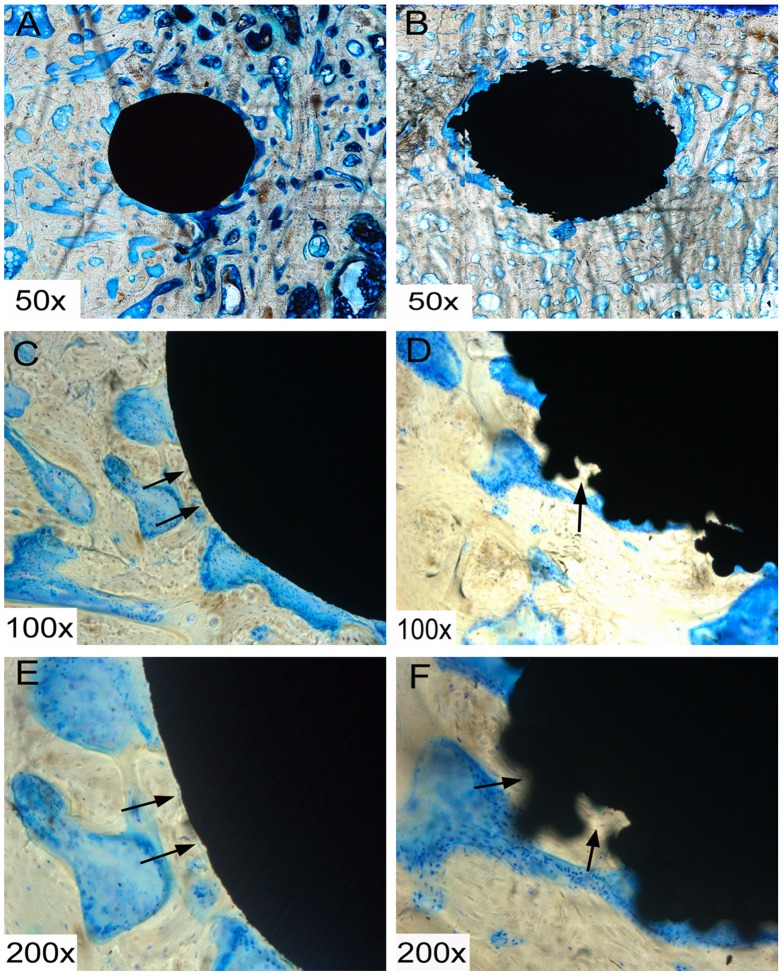
Histological sections of smooth surface (SS) (A, C and E) and roughened surface (RS) (B, D and F) screws at 12 weeks. Fibrous tissue, which was evident at 6 weeks, had almost disappeared by this timepoint. Black arrows indicate that the bone tissue integrates with the screws and grows into the gaps of the RS.

After 6 weeks of implantation, fibrous tissue formation could be seen around the RS and SS screws. By 12 weeks, this fibrous tissue formation had been mostly replaced with newly formed bone (Figs. 4 and 5), which was observed to make contact with the screw at the bone-screw interface, and newly formed bone grew into the gaps on the RS surface, but not into SS screws.

### Three-dimensional Imaging and Reconstruction

An ROI surrounding the RS and SS screws was reconstructed and analyzed using the same thresholding conditions. At 12 weeks, the bone volume/total volume (BV/TV; 82% ±2%), bone surface area/bone volume (BS/BV; 4.51±0.1 mm^−1^) and trabecular number (Tb.N; 1.89±0.03 mm^−1^) within the ROIs of the RS screws were significantly higher than those of the SS screws (BV/TV, 73%±5%; BS/BV, 4.18±0.67 mm^−1^; Tb.N, 1.61±0.12 mm^−1^; P<0.05; Table 1). Furthermore, Tb.N (1.89±0.03 mm^−1^) for the RS screw ROI at 12 weeks was significantly greater than that observed for the RS screw ROI at 6 weeks (1.53±0.06 mm^−1^; P<0.05).

**Table 1:** ROI parameters of the SS and the RS screws (mean±SD).

Parameter		SS (6 weeks)	RS (6 weeks)	SS (12 weeks)	RS (12 weeks)
BV/TV (%)		72±4	76±6	73±5	82±2
BS/BV (mm^−1^)		4.13±0.07	4.32±0.2	4.18±0.67	4.51±0.1
Tb.Th (mm)		0.44±0.04	0.49±0.06	0.45±0.02	0.47±0.04
Tb.N (mm^−1^)		1.56±0.12	1.53±0.06	1.61±0.12	1.89±0.03

BV/TV: Bone volume/Total volume; BS/BV: Bone surface area/Bone volume; Tb.Th: Trabecular thickness. Tb.N: Trabecular number.

### Mechanical Testing

**Figure 6:**
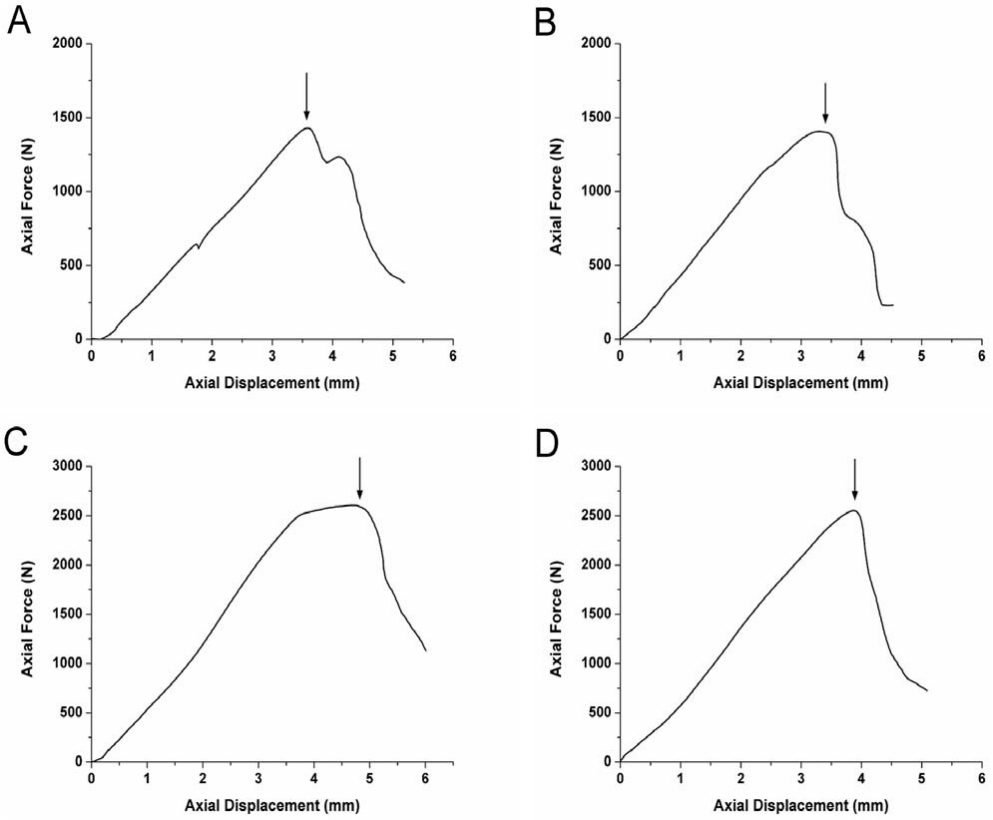
Pull-out test curves of smooth surface (SS) and roughened surface (RS) screws at (A and B) 6 weeks and (C and D) 12 weeks. Black arrows indicate the highest load that the bone–screw interface could resist before failure.

**Figure 7:**
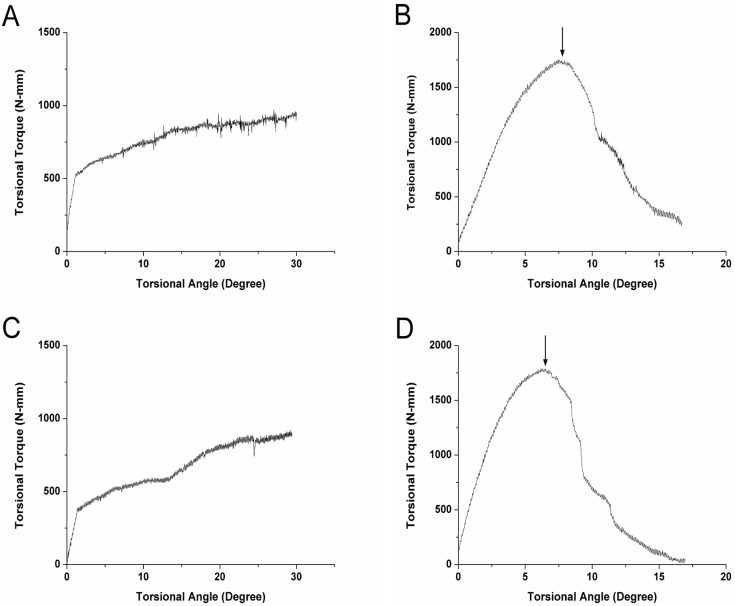
Torsion test curves of smooth surface (SS) and roughened surface (RS) screws at (A and B) 6 weeks and (C and D) 12 weeks. Black arrows indicate where the RS fractured in relation to the stress.

**Figure 8:**
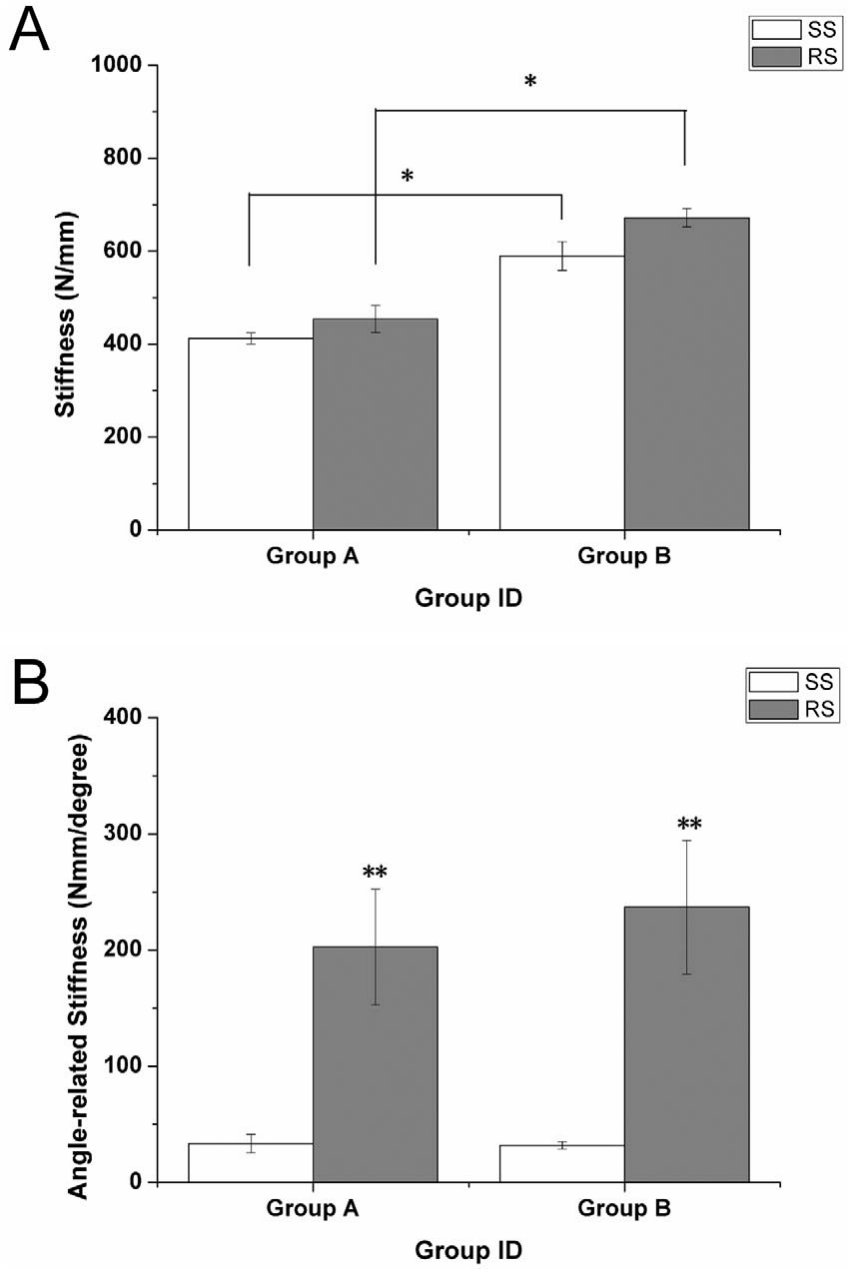
(A) Stiffness and (B) angle-related stiffness at 6 (group A) and 12 weeks (group B).

**Figure 9:**
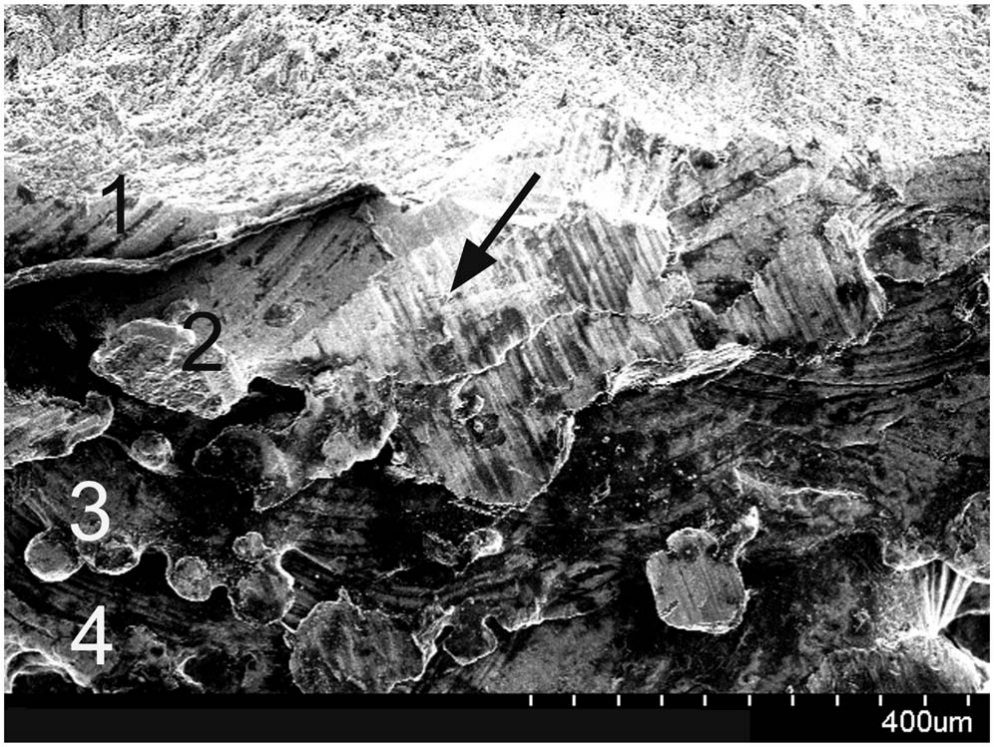
Scanning electron microscopy image of the broken end of roughened surface (RS) screw; each layer of the melted Ti6Al4V (No 1–4) and the broken end (black arrow) can be observed clearly.

A significant increase was noted in the maximum pullout strength required as the in situ duration of the screw increased (1429.67±28.09 N at 6 weeks *vs.* 2555.03±71.08 N at 12 weeks in the SS group; and 1470.1±59.13 N at 6 weeks *vs.* 2668.38±107.66 N at 12 weeks for the RS group; P<0.05; Fig. 6); however, no significant difference was observed between the two groups at either time point. At both 6 weeks and 12 weeks, respectively, the maximum torque required in the RS group (1729.25±68.07 Nmm and 1753.67±63.60 Nmm, respectively) was significantly higher than that in the SS group (941.15±41.29 Nmm and 943.88±53.25 Nmm, respectively; P<0.05; Fig. 7). Similar differences were found for measurements of stiffness and angle-related stiffness between the RS and SS groups at both the 6- and 12-week time points (Fig. 8). For the torsion tests, the RS screws fractured following rotation of 6–7°, whereas SS screws failed at a lower degree of rotation. The fractured end of one of the RS screws is shown in Figure 9. The image also shows each layer of the melted Ti_6_Al_4_V (No 1–4) and the broken end (black arrow). The results of mechanical testing are summarized in table 2.

**Table 2:** Mechanical testing results of pullout tests and torsion tests (mean±SD).

Parameter	SS (6 weeks)	RS (6 weeks)	SS (12 weeks)	RS (12 weeks)
Maximum pullout strength (N)	1429.67±28.09	1470.1±59.13	2555.03±71.08*	2668.38±107.66*
Maximum torque (Nmm)	941.15±41.29	1729.25±68.07*	943.88±53.25	1753.67±63.60*
Stiffness (N/mm)	412.35±12.04	454.12±29.24	589.57±30.70*	671.82±20.01*
Angle-related stiffness (Nmm/degree)	33.33±7.83	202.48±49.87*	31.77±3.12	236.90±57.42*

*For the maximum pullout strength and stiffness, significant difference was observed between SS and RS in the same group, and for the maximum torque and angle-related stiffness, there was significant difference between the two groups at either time point.

## Discussion

Screw loosening still constitutes a significant problem in orthopedics [17]. Traditionally, cement augmentation or expandable screws have been used to improve implant fixation. However, “cement disease” caused by osteolysis can result in implant loosening, and expandable screws can only strengthen the local mechanical property of the tissue and are thus potentially inadequate for osteoporosis patients where bone is already much weaker. Thus, cementless techniques continue to be improved with the aim of improving and augmenting fixation. Broadly speaking, cementless techniques are those that modify the implant surface morphology, referred to as ‘surface coatings’, which includes techniques such as plasma-sprayed coatings, acid etching, and other electrochemical processes, or grit blasting with abrasives [18], [19]. A variety of materials may be modified by these surface coating methods. Ti_6_Al_4_V is a medical material that is considered to have high biocompatibility and is therefore routinely used in bone repair strategies [20]. Most custom titanium implants are fabricated by machining, casting, and the various surface coatings described above [21], [22]. However, direct metal fabrication, such as EBM, offers advantages over these technologies because it not only changes the implant surface morphology directly, but it can be used to fabricate implants with specific shape and structure, according to computer-aided design (CAD) files.

Haslauer and co-workers [23] assessed the biocompatibility of human adipose-derived adult stem cells on EBM Ti_6_Al_4_V discs as compared with Ti_6_Al_4_V discs from a commercial source. They found that a porous EBM structure supported increased cell proliferation without an increase in IL-6 and IL-8 expression, indicating better biocompatibility between the cells and the EBM-fabricated specimens. Other *in vitro* studies have also shown that rough titanium can improve mesenchymal stem cell differentiation [24]–[26]. It is widely accepted that a RS is also an effective way to promote implant stability [27]–[29]. A rougher surface not only improves friction between the host bone and the implant, but allows bone growth into the gaps on the surfaces of the scaffold, thus promoting bone-implant anchorage. Indeed, our SEM and histological analyses (Figs. 3, 4, and 5) showed increased bone ingrowth in the RS surfaces. Hansson and Norton [30] observed that half-spherical micropits achieved the greatest cell retentive capacity and that a diameter of 1–5 µm was the optimal size for these pits; the surface characteristics of the RS surface in our study were consistent with this conclusion. The roughened structure also increases the surface area of the implants. Thus, compared with smooth surfaced implants with a similar shape and fabricated with the same material, those with a rougher surface will show improved adhesion to bone tissue and provide increased stability. Our histological staining indicated an increase in tissue production around the RS screws, and our three-dimensional micro-CT imaging results confirmed this speculation, with an increased bony surface area for RS screws and an enhanced implant-bone contact area.

For the pullout tests, we observed no significant difference between the SS and RS screws at both 6 and 12 weeks, although maximum pullout strength did increase significantly over time. We speculated that this lack of difference might be because the SS and RS screws have a similar shape, size, pitch, and thread depth. Previous studies have shown that maximum pullout strength can be affected by changing the screw shape. Kim et al. [31] evaluated fixation strength using screws with different thread shapes and found that, regardless of bone density, screws with an inner conical and outer cylindrical configuration and a V-shaped thread produced the maximum pullout strength. Choi and co-workers [32] also showed that pullout strength was based on screw thread shape, with a V-shaped design showing the largest strength, followed by a square and then a buttress design. Gao et al. [33] evaluated the fixation strength of conventional and expansive pedicle screws (CPS and EPS) and found that, compared with the CPS group, the maximum pullout strength in the EPS group was increased by 18.2%, 36.5%, 27.2%, and 51.5% in bone samples determined as normal, osteopenic, osteoporotic, and severely osteoporotic, respectively, according to bone mineral density measurements.

For the torsion tests, the RS screws fractured when they were rotated to approximately 6–7°, and the corresponding torque at fracture was greater than the maximum torque withstood by SS screws, indicating that bone grew into the gaps of the RS screws thereby increasing the resistance to torque. As shown in Figure 9, the fracture occurred along the bone-implant interface, which is a zone that contains stress concentrations. In addition, the image shows each layer of the melted Ti_6_Al_4_V (No 1–4), which is a limitation of using EBM for fabrication. EBM produces metal implants with melted metal powder in a layer-by-layer fashion; thus the binding between adjacent layers will not be as tight in this type of screw and may lead to implant fracture. This drawback can be remedied by fabricating a compact structure for bone-implant interfaces. Indeed, we believe that the stability of the screws can be improved by increasing the area of the bone-implant interface in addition to changing its shape to improve the long-term stability of the screws.

Our findings should be interpreted within the context of the study limitations. In this investigation, we only evaluated the histological and biomechanical properties of the roughened titanium screws fabricated by EBM. Thus, further studies concerning the relationship between screw structure and its mechanical properties should be performed. In addition, future studies are needed to ascertain if there are any adverse effects of residual Ti_6_Al_4_V powder in repair sites.

## Conclusion

EBM is a simple and effective method for producing rough-surfaced screws. After 12 weeks, RS screws demonstrated a higher degree of osseointegration and show better anti-torsion properties than SS screws. This study highlights the potential of fabricating rough-surfaced screws by EBM, obviating the need for additional surface modification.

## References

[1] Wu JC, Huang WC, Tsai HW, Ko CC, Wu CL, et al (2011) Pedicle screw loosening in dynamic stabilization: incidence, risk, and outcome in 126 patients. Neurosurg Focus 4: E9. 10.3171/2011.7.FOCUS11125 21961872

[2] Ko CC, Tsai HW, Huang WC, Wu JC, Chen YC, et al (2010) Screw loosening in the Dynesys stabilization system: radiographic evidence and effect on outcomes. Neurosurg Focus 6: E10. 10.3171/2010.3.FOCUS1052 20568916

[3] Mario DS, Patrizio P, Francesco L, Georgios B (2007) Complications of thoracic pedicle screws in scoliosis treatment. Spine 15: 1655–1661. 10.1097/BRS.0b013e318074d604 17621214

[4] Yusuf KD, Mesut M, Baha Z, Mehmet S (2013) Case Report Missing screw as a rare complication of anterior cervical instrumentation. Case Reports in Orthopedics. 10.1155/2013/593905 PMC3619545 23634313

[5] Anselme K, Linez P, Bigerelle M, Le MD, Le MA, et al (2000) The relative influence of the topography and chemistry of TiAl6V4 surfaces on osteoblastic cell behaviour. Biomaterials 21: 1567–1577. 10885729 10.1016/s0142-9612(00)00042-9

[6] Sammons RL, Lumbikanonda N, Gross M, Cantzler P (2005) Comparison of osteoblast spreading on micro-structured dental implant surfaces and cell behaviour in an explant model of osseointegration. A scanning electron microscopic study. Clin Oral Implants Res 16: 657–666. 16307572 10.1111/j.1600-0501.2005.01168.x

[7] Bowers KT, Keller JC, Randolph BA, Wick DG, Michaels CM (1992) Optimization of surface micromorphology for enhanced osteoblast responses in vitro. Int J Oral Maxillofac Implants 7: 302–310. 1289255

[8] Jenny B, Thomas H, Falko S, Nicholas D, Spencer HH (2012) Response of osteoclasts to titanium surfaces with increasing surface roughness: An In vitro study. Biointerphases 7: 34. 22639093 10.1007/s13758-012-0034-x

[9] Yang J, Wang J, Yuan T, Zhu XD, Xiang Z, et al (2013) The enhanced effect of surface microstructured porous titanium on adhesion and osteoblastic differentiation of mesenchymal stem cells. J Mater Sci: Mater Med 9: 2235–2246. 10.1007/s10856-013-4976-4 23779154

[10] Zhao C, Zhu X, Liang K, Ding J, Xiang Z, et al (2010) Osteoinduction of porous titanium: a comparative study between acid-alkali and chemical–thermal treatments. J Biomed Mater Res B Appl Biomater 95B: 387–396. 10.1002/jbm.b.31728 20878923

[11] Takemoto M, Fujibayashi S, Neo M, Suzuki J, Matsushita T, et al (2006) Osteoinductive porous titanium implants: effect of sodium removal by dilute HCl treatment. Biomaterials 27: 2682–2691. 16413052 10.1016/j.biomaterials.2005.12.014

[12] Yu S, Yu Z, Wang G, Han J, Ma X, et al (2011) Preparation and osteoinduction of active micro-arc oxidation films on Ti-3Zr-2Sn-3Mo-25Nb alloy. Trans Nonferrous Met Soc China 21: 573–580.

[13] Shiyong W, Wei L, Zixiang W, Da L, Mingxuan G, et al (2010) Biomechanical and histological evaluation of an expandable pedicle screw in osteoporotic spine in sheep. Eur Spine J 19: 2122–2129. 20577766 10.1007/s00586-010-1489-4 PMC2997211

[14] Cook SD, Barbera J, Rubi M, Salkeld SL, Whitecloud TS III (2001) Lumbosacral fixation using expandable pedicle screws: an alternative in reoperation and osteoporosis. Spine J 1: 109–114. 14588390 10.1016/s1529-9430(01)00020-1

[15] Cook SD, Salkeld SL, Whitecloud TS III, Barbera J (2000) Biomechanical evaluation and preliminary clinical experience with an expansive pedicle screw design. J Spinal Disord 13: 230–236. 10872761 10.1097/00002517-200006000-00006

[16] Panjabi MM (1988) Biomechanical evaluation of spinal fixation devices: I. A conceptual framework. Spine 10: 1129–1134. 10.1097/00007632-198810000-00013 3206270

[17] Ohlin A, Karlsson M, Duppe H, Hasserius R, Redlund-Johnell I (1994) Complications after transpedicular stabilization of the spine. A survivorship analysis of 163 cases. Spine (Phila Pa 1976) 19: 2774–2779. 7899978 10.1097/00007632-199412150-00007

[18] Le Guéhennec L, Soueidan A, Layrolle P, Amouriq Y (2007) Surface treatments of titanium dental implants for rapid osseointegration. Dent Mater 7: 844–854. 10.1016/j.dental.2006.06.025 16904738

[19] Schliephake H, Scharnweber D (2008) Chemical and biological functionalization of titanium for dental implants. J. Mater. Chem 18: 2404–2414.

[20] Van Noort R (1987) Titanium: the implant material of today. J Mater Sci 22: 3801–3811.

[21] Karageorgiou V, Kaplan D (2005) Porosity of 3D biomaterial scaffolds and osteogenesis. (2005). Biomaterials 26: 5474–5491. 15860204 10.1016/j.biomaterials.2005.02.002

[22] Lopez-Heredia MA, Goyenvalle E, Aguado E, Pilet P, Leroux C, et al (2008) Bone growth in rapid prototyped porous titanium implants. J Biomed Mater Res A 85A: 664–673. 10.1002/jbm.a.31468 17876801

[23] Haslauer CM, Springer JC, Harrysson OL, Loboa EG, Monteiro-Riviere NA, et al (2010) In vitro biocompatibility of titanium alloy discs made using direct metal fabrication. Medical Engineering & Physics 32: 645–652. 20447856 10.1016/j.medengphy.2010.04.003

[24] Olivares-Navarrete R, Hyzy SL, Hutton DL, Erdman CP, Wieland M, et al (2010) Direct and indirect effects of microstructured titanium substrates on the induction of mesenchymal stem cell differentiation towards the osteoblast lineage. Biomaterials 31: 2728–2735. 20053436 10.1016/j.biomaterials.2009.12.029 PMC2821717

[25] Hu Y, Cai K, Luo Z, Zhang R, Yang L, et al (2009) Surface mediated in situ differentiation of mesenchymal stem cells on gene-functionalized titanium films fabricated by layer-by-layer technique. Biomaterials 30: 3626–3635. 19371947 10.1016/j.biomaterials.2009.03.037

[26] Cai K, Lai M, Yang W, Hu R, Xin R, et al (2010) Surface engineering of titanium with potassium hydroxide and its effects on the growth behavior of mesenchymal stem cells. Acta Biomater 6: 2314–2321. 19963080 10.1016/j.actbio.2009.11.034

[27] Le Guéhennec L, Soueidan A, Layrolle P, Amouriq Y (2006) Surface treatments of titanium dental implants for rapid osseointegration. Dental materials 23: 844–854. 16904738 10.1016/j.dental.2006.06.025

[28] Aparicio C, Padrós A, Gil FJ (2011) In vivo evaluation of micro-rough and bioactive titanium dental implants using histometry and pull-out tests. J Mech Behav Biomed Mater 4: 1672–1682. 22098868 10.1016/j.jmbbm.2011.05.005

[29] Cho SA, Park KT (2003) The removal torque of titanium screw inserted in rabbit tibia treated by dual acid etching. Biomaterials 24: 3611–3617. 12809791 10.1016/s0142-9612(03)00218-7

[30] Hansson S, Norton M (1999) The relation between surface roughness and interfacial shear strength for bone-anchored implants. A mathematical model. J Biomech 32: 829–836. 10433425 10.1016/s0021-9290(99)00058-5

[31] Kim YY, Choi WS, Rhyu KW (2012) Assessment of pedicle screw pullout strength based on various screw designs and bone densities–an ex vivo biomechanical study. The Spine Journal 12: 164–168. 22336467 10.1016/j.spinee.2012.01.014

[32] Choi W, Lee S, Kim JW (2002) Assessment of pullout strengths of various pedicle screw designs in relation to the changes in the bone mineral density. Paper presented at 49th Annual Meeting of the Orthopedic Research Society; February 10–13, Dallas, TX.

[33] Gao M, Lei W, Wu Z, Liu D, Shi L (2011) Biomechanical evaluation offixation strength of conventional and expansive pedicle screws with or without calcium based cement augmentation. Clinical Biomechanics 26: 238–244. 21084138 10.1016/j.clinbiomech.2010.10.008

